# Neutralization of mouse interleukin-17 bioactivity inhibits corneal allograft rejection

**Published:** 2011-08-11

**Authors:** XueDong Chen, ShiYong Zhao, XianLing Tang, HongYan Ge, Ping Liu

**Affiliations:** 1 Eye Hospital, The First Affiliated Hospital of Harbin Medical University, Harbin, China; 2 Department of Hepatopancreatobiliary Surgery, The Second Affiliated Hospital of Harbin Medical University, Harbin, China

## Abstract

**Purpose:**

To investigate the inhibitory effects of anti-mouse interleukin-17 (IL-17) monoclonal antibody (mAb) in high-responder corneal allograft rejection.

**Methods:**

C57BL/6 or BALB/c mice corneal grafts were grafted onto BALB/c hosts. The neutralizing mouse IL-17 antibody and isotype control were injected intraperitoneally immediately after transplantation for experimental treatment. At appropriate times after treatment, recipient grafts were assessed clinically and histologically, and recipient corneal graft- infiltrating cells were detected by immunohistochemistry and quantified by real-time PCR. The cytokine spleen levels of T helper type 1 (Th1), Th2, and Th17 were analyzed by enzyme-linked immunosorbent assay. Flow cytometric analysis was used to evaluate the frequencies of IL-17-producing Th17 cells.

**Results:**

Neutralization of IL-17 with anti-IL-17 mAb obviously prolonged allograft survival compared to the group that received isotype control. Neovascularizations and inflammatory immune cells in corneal stroma decreased in the allogeneic recipients treated with anti-IL-17 mAb. The mRNA (mRNA) level of graft-infiltrating cells, including neutrophiles, cluster of differentiation 4 (CD4) T cells, and CD8 T cells, decreased dramatically in the IL-17 neutralization group. At days 14 and 42, splenocytes from recipients treated with anti-IL-17 mAb produced significantly less of the pro-inflammatory cytokines interferon-gamma (IFN-γ), IL-12p40, and IL-17 compared to those from control Ig-treated recipients at day 14. However, Th2 cytokine IL-4 and IL-5 production increased, and IL-13 levels were not significantly different among the three groups. IL-6 production was elevated in recipients treated with anti-IL-17 mAb. Anti-IL-17 mAb reduced the percentage of Th17 in CD4^+^ T cells, but there was no statistical significance between anti-IL-17 mAb and the control group.

**Conclusions:**

Neutralization of mouse IL-17 bioactivity with anti-IL-17 mAb improves allogeneic corneal graft survival and inhibits corneal allograft rejection to a certain extent by inhibiting production of graft-infiltrating inflammatory cells and decreasing the secretion of pro-inflammatory cytokines.

## Introduction

Corneal allografts enjoy high rates (40%–50%) of spontaneous acceptance compared with other types of transplantation [1]. Allograft rejection is the main cause of corneal graft failure. The 5-year survival rate of low-risk keratoplasty is approximately 90%, even without human leukocyte-antigen matching [2]. In contrast, the survival rate of high-risk keratoplasty decreases significantly to below 50% due to immune-mediated rejection [3,4]. Allograft rejection is histologically characterized by a massive infiltration of T cells, especially cluster of differentiation 4 (CD4) T cells, which play an important role in the response to allogeneic corneal cells [5]. Existing information [6-8] on the molecular mechanisms governing the interactions between immunocompetent cells indicates that cytokines play an important role in the maintenance of graft inflammation, tissue destruction, and rejection.

Both T helper type 1 (Th1) and Th2 responses in acute allograft rejection have been investigated. Th1 cells, which mediate rejection, are mainly associated with mononuclear cell infiltration of the grafts, and they characteristically secrete interferon-gamma (IFN-γ) and express transcription factor T-bet (T-bet). Th2 cells, which are involved in inducing transplantation tolerance, are generally related to eosinophil infiltration of the grafts and produce interleukin-4(IL-4), IL-5, and IL-13 [9-12]. Recently, the Th1/Th2 paradigm has been challenged by the finding that Th17 may participate in transplant immunity. Th17 cells produce large amounts of IL-17, IL-17 F, IL-21, and IL-22. In addition, transforming growth factor beta (TGF-β), IL-6, and IL-21 may induce naive T cells to differentiate into Th17 cells under the influence of the orphan nuclear receptor, retinoid related orphan receptor gammat (RORγt) [13].

IL-17 is a potent pro-inflammatory cytokine that induces chemokine expression and leukocyte infiltration and mediates tissue inflammation [14]. IL-17 has been implicated in allograft rejection of renal [15,16], cardiac [17,18], lung [8,19-21], and vascular [22] tissues. Many recent studies have focused on the effect of IL-17 antagonists on allograft rejection. It was reported that an IL-17 antagonist prolonged nonvascularized and vascularized cardiac allograft median survival time [23], and IL-17 neutralization inhibited accelerated cardiac allograft rejection in a model of chronic allograft vasculopathy in T-bet^−/−^ mice [24]. IL-17 antagonism inhibits acute but nonchronic vascular rejection [22]. However, little is known about the therapeutic efficacy of IL-17 neutralization in acute murine corneal allograft rejection.

## Methods

### Mice and anesthesia

Animals were 6- to 8-week-old female BALB/c and C57BL/6 mice provided by the Experimental Animal Center of the First Affiliated Hospital of Harbin Medical University (Harbin, China), and all animal procedures were approved by the animal care board. Animals were treated according to the Association for Research in Visio and Ophthalmology Statement on the Use of Animals in Ophthalmic and Vision Research. Each animal was deeply anesthetized by intraperitoneal injection of 3 to 4 mg of ketamine and 0.1 mg of xylazine.

### Corneal transplantation and the evaluation of graft survival

Penetrating keratoplasty in mice has been described previously [25]. Briefly, C57BL/6 or BALB/c donor corneal grafts with 2-mm diameters were excised and placed in the same sized BALB/c recipient beds with eight interrupted 11–0 nylon sutures (Huawei, Hangzhou, China). The grafts were examined by slit-lamp biomicroscopy twice a week and scored using a previously described scoring system [26]. Briefly, grafts that received an opacity score of 2^+^ or greater (mild, deep stromal opacity with pupil margin, and iris vessels visible) at 3 weeks or 3^+^ or greater (moderate stromal opacity with only the pupil margin visible) at 2 weeks after transplantation were considered rejected (immunologic failure).

### Treatment with antibody

The neutralizing mouse IL-17 mAb and isotype control (R&D systems, Minneapolis, MN) were used for treatment. The recipients were injected intraperitoneally with 0.1 mg anti-IL-17 mAb or isotype control daily on days 0–3, followed by every other day until day 13 after transplantation [24].

### Histologic analysis

Recipients grafts were fixed and frozen in liquid nitrogen. The 4-µm frozen sections were fixed in acetone for 10 min and air dried. Slides were stained with rat antimouse Ly6G (neutrophils), CD4 (CD4^+^ T cells), and CD8 (CD8^+^ T cells) antibodies (Santa Cruz Biotechnology, Santa Cruz, CA), then with goat antirat FITC (ZSGB-BIO, BeiJing, China) as secondary antibodies. FITC-conjugated rat antimouse CD11b antibody (BD PharMingen, San Diego, CA) was used to detect the recruitment of macrophages. For histologic assessment, corneal grafts were fixed in 10% neutral-buffered formalin and 4-µm sections were routinely stained by hematoxylin and eosin.

### Quantitative real-time PCR

The mRNA level of granulocyte differentiation antigen 1 (*Gr-1*; neutrophils), macrophage differentiation antigen-1 (*Mac-1*; macrophages), *CD4* (CD4^+^ T cells), and *CD8* (CD8^+^ T cells) in recipients grafts was detected by real-time PCR. Total RNA was extracted from corneal tissue, using the TRIZOL reagent (Invitrogen, Foster City, CA). Total RNA was quantified using an ultraviolet spectrophotometer, and 0.5 μg of total RNA was subsequently processed to cDNA by reverse transcription. Real-time PCR was performed on cDNA. Primer-binding DNA was detected by EVA green dye (BioRad, Hercules, CA). Relative expression of the gene of interest was expressed as the comparative concentration of the product compared with β-actin (*Actb*) product, as calculated by the MxPro QPCR software (Agilent Technologies, Santa Clara, CA).

### Enzyme-linked immunosorbent assay (ELISA)

Spleens from recipients of corneal grafts were harvested for ELISA. The supernatant was collected, and IFN-γ, IL-4, IL-5, IL-6, IL-12p40, IL-13, and IL-17 production was determined according to the manufacturer’s instructions. The levels of IFN-γ and IL-17 in spleens were quantified using the ELISA kit for murine IL-17 and IFN-γ (R&D systems). IL-4, IL-5, IL-6, IL-12p40, and IL-13 production was quantified using murine IL-4, IL-5, IL-6, IL-12p40, and IL-13 antibody pairs for ELISA (BD PharMingen). All measurements were performed in duplicate.

### Flow cytometry

Spleens were retrieved, mashed, and then erythrocytes were lysed. Cells were stained extracellularly with antimouse CD3 PerCP-Cy5.5, antimouse CD4 FITC, and appropriate isotype control mAb (eBioscience, San Diego, CA) as per the manufacturer’s instructions. Splenocytes were fixed and permeabilized with fixation/permeabilization solution (BD PharMingen) according to the manufacturer’s instructions and then stained intracellularly with antimouse IL-17 APC and appropriate isotype control mAb (eBioscience) in the dark for 30 min. Data collection and analysis were performed on a FACS Calibur flow cytometer using CellQuest software (BD Bioscience).

### Statistics

For graft survival, the log-rank test was used to calculate p values. For other experiments, data were calculated as mean±SEM, and Student *t* test was used to compare mean values. All statistical analyses were performed using SPSS 13.0 software (SPSS, Chicago, IL). A p value <0.05 was considered statistically significant.

## Results

### Neutralizing interleukin-17 antibody delayed corneal allograft rejection

**Figure 1:**
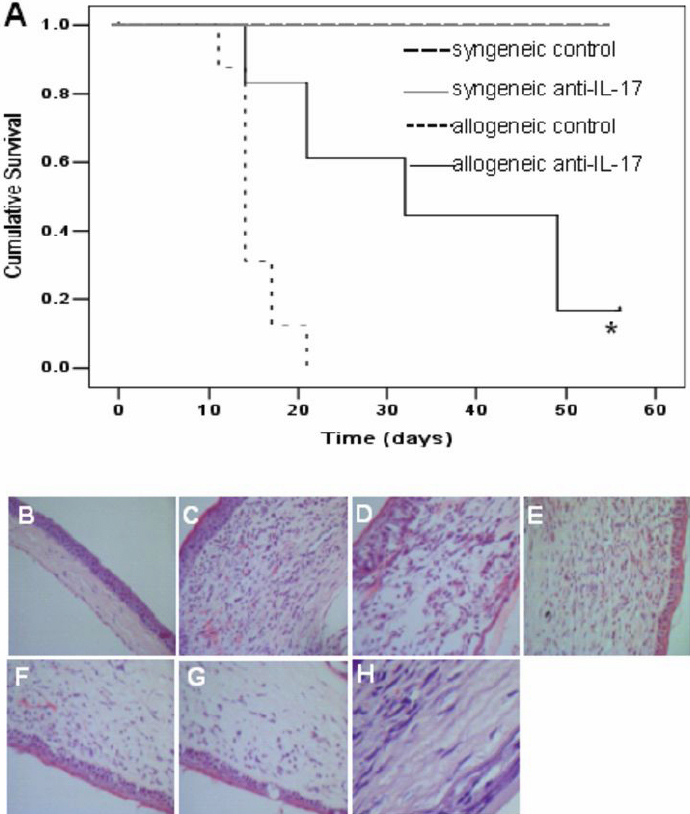
Interleukin-17 neutralization delayed corneal allograft rejection.

We administered anti-IL-17 mAb or control Ig to syngeneic and allogeneic recipients and observed the grafts to determine the effect of IL-17 neutralization on graft survival time. Our results showed that all of the corneal syngenic grafts survived for 56 days after transplantation and that neutralization of IL-17 with anti-IL-17 mAb did not affect the survival time. However, almost all of the corneal allografts were rejected at 20 days after transplantation in the control group. Results from Kaplan–Meier survival curves showed that neutralization of IL-17 with anti-IL-17 mAb indeed prolonged allograft survival time compared with the group that received control IgG (Figure 1A). Pathological results showed that the normal corneal endothelium was intact and that no inflammatory cells existed in corneal stroma. However, we found that the corneal stroma were edema, and inflammatory cells were present in thickened stroma after syngeneic or allogeneic transplantation. Moreover, massive neovascularizations were found in allogeneic corneal stroma. In syngeneic groups, the edema in cornea from IL-17 antibody-treated recipients was improved compared to edema in cornea from control Ig-treated recipients after transplantation. In contrast, corneal endothelium of grafts from allogenic recipients treated with IL-17 antibody was more intact and neovascularization and inflammatory cells in the stroma decreased significantly compared to control Ig-treated recipients (Figure 1B-H).

### Neutralizing interleukin-17 antibody inhibited graft-infiltrating cell expression after corneal allogeneic transplantation

**Figure 2:**
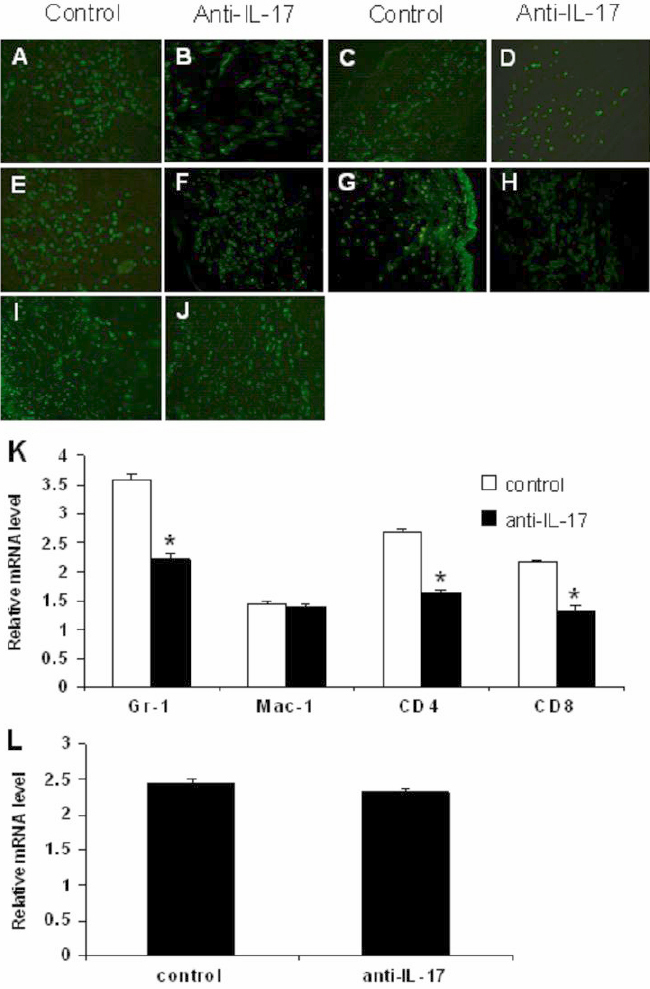
Interleukin-17 neutralization inhibited graft-infiltrating cells expression at day 14 after corneal allogeneic transplantation.

Massive inflammatory cells were present in the corneal stroma after transplantation. Neutrophils, macrophages, CD4^+^ T cells, and CD8^+^ T cells were predominant in corneal allografts at 2 weeks after surgery, and neutrophils were mainly in corneal syngeneic grafts (Figure 2A-J). To determine whether the delayed allograft rejection in the group treated with anti-IL-17 mAb correlated with decreased infiltration of inflammatory cells, the mRNA level of graft-infiltrating cells was detected by real-time PCR. We found that *Gr-1*, *CD4*, and *CD8* expression in the inflamed allografts of anti-IL-17 mAb treatment recipients (2.22±0.1, 1.64±0.04, and 0.04±0.1, respectively) decreased significantly compared to those of control recipients (3.61±0.08, 2.69±0.06, and 2.17±0.04, respectively; p<0.01) on day 14 after transplantation. In contrast, *Mac-1* expression did not alter appreciably (1.39±0.06 versus 1.44±0.07; p=0.519). In syngeneic corneal grafts treated with anti-IL-17 mAb, *Gr-1* expression did not decrease significantly compared to control grafts on day 14 after transplantation (2.32±0.05 versus 2.44±0.06; p=0.141; Figure 2K-L).

### Neutralizing interleukin-17 antibody inhibited pro-inflammatory cytokine expression after corneal allogeneic transplantation

**Figure 3:**
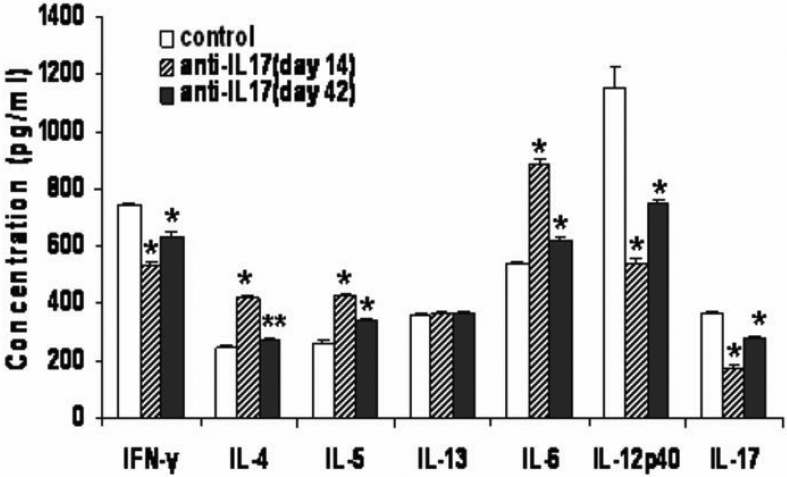
Interleukin-17 neutralization inhibited proinflammatory cytokine expression after corneal allogeneic transplantation.

We examined the production of Th1-, Th2-, and Th17-related cytokines in the anti-IL-17 mAb or control Ig-treated recipients of corneal spleens 14 or 42 days after transplantation. We found that at days 14 and 42, splenocytes from allogeneic recipients treated with anti-IL-17 mAb produced significantly less pro-inflammatory cytokines compared to control Ig-treated recipients at day 14 (IFN-γ [529.8±13.83 and 636.5±9.34 versus 741.48±10.51, respectively], IL-12p40 [539.58±10.74 and 750.62±8.00 versus 1156.9±69.93], and IL-17 [173.7±8.11 and 273.6±10.08 versus 366.13±7.93; p<0.01]). In contrast, Th2 cytokines IL-4 (420.25±4.47 and 267.76±5.34 versus 248.69±5.67, respectively; p<0.05) and IL-5 (425.38±5.91 and 343.5±6.14 versus 262.06±5.27; p<0.01) production increased in splenocytes from recipients treated with anti-IL-17 mAb compared to the control Ig-treated recipients. However, IL-13 levels (367.48±5.05 and 363.74±6.55 versus 359.87±4.81; p=0.31 and p=0.65, respectively) were not significantly different from the control group. IL-6 (887.17±16.36 and 620.28±11.09 versus 537.35±12.01, respectively; p<0.01) production was elevated in recipients treated with anti-IL-17 mAb (Figure 3). Interestingly, the above cytokines were not found in splenocytes from syngeneic recipients treated with anti-IL-17 mAb or control IgG at days 14 and 42 after transplantation.

### Neutralizing interleukin-17 antibody did not reduce the percentage of CD4^+^ Th17 cells

**Figure 4:**
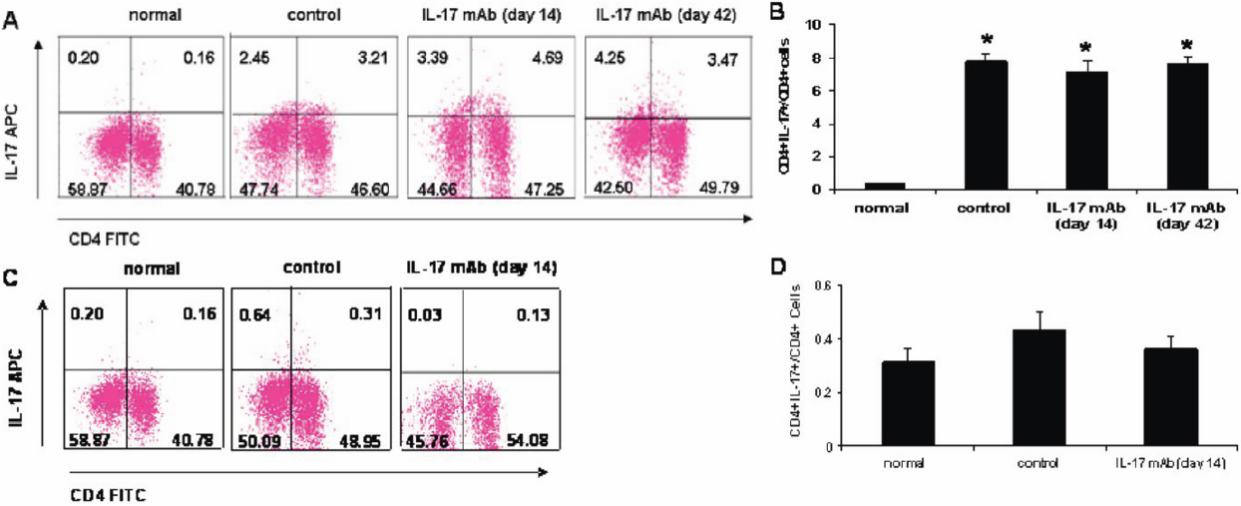
The percentages of CD4+Th17 cells in spleens.

For the allogeneic recipients, the percentage of CD4^+^ IL-17^+^ in CD4^+^ cells in the isotype control, anti-IL-17 mAb (day 14), and anti-IL-17 mAb (day 42) groups (7.73±0.55%, 7.12±0.64%, and 7.63±0.46%, respectively) increased markedly compared with the normal group (0.31±0.05%; p<0.01). Although the percentage of CD4^+^ IL-17^+^ in CD4^+^ cells in the anti-IL-17 mAb group trended lower than that of the isotype control group, there were no significant differences among them (p>0.05). In the syngeneic recipients, there were no significant differences in the isotype control (0.43±0.07%), anti-IL-17 mAb (0.36±0.05%), and normal group (0.31±0.05%) (p=0.17 and p=0.41, respectively; Figure 4A-B).

## Discussion

Although many studies have demonstrated the effect of IL-17 on allograft rejection, controversy still exists regarding the role of IL-17 in corneal transplant rejection. A pathogenic role of IL-17 at the early stage of corneal allograft rejection has been described by Chen et al. [27], while Yamada et al. [28] thought that IL-17 did not have a critical role in the development of minor specific allograft rejection in C57BL/6 mice. However, Cunnusamy et al. [29] argued that IL-17A is not required for corneal allograft rejection and may instead contribute to the immune privilege of corneal allografts. Our study supports the pathogenic role of IL-17 in corneal allograft rejection. This discrepancy may be attributed to different timing when the target gene was detected and a different model of corneal transplantation. In our study, we demonstrated the effects of antimouse IL-17 antibody treatment on corneal allograft rejection at 14 and 42 days after transplantation in a wild-type murine corneal transplant model instead of an IL-17 knockout murine keratoplasty model as shown by other reports. We then analyzed the relationships of delayed allograft rejection with decreased infiltration of inflammatory cells and pro-inflammatory cytokines.

Our results showed that neutralizing IL-17 antibody significantly improved the survival rate of corneal allogeneic transplantation but had no effect on the survival time of syngeneic grafts. Consistently, histological analysis of corneal allografts revealed that neutralizing IL-17 antibody decreased tissue damage and decreased graft-infiltrating neutrophil, CD4^+^ T-cell, and CD8^+^ T-cell expression. We found that neutrophils increased due to inflammation during the early postoperative period, and CD4^+^ and CD8^+^ T cells mediated the T-cell response in rejecting mice due to the adaptive immune response. Neutrophil depletion by anti-Gr1 antibody injections significantly delayed MHC class II incompatible skin allograft rejection in IL-4-deficient recipients [30]. Similarly, neutrophil depletion delayed cardiac allograft rejection in IFN-deficient mice [31]. Anti-CD4/CD8 mAbs greatly reduced the prevalence of cardiac allograft vasculopathy and eliminated the development in mice rendered tolerant by the induction of mixed chimerism [32]. These findings suggest that neutrophils, CD4^+^ T cells, and CD8^+^ T cells play an important role in allograft rejection. Furthermore, the results of mRNA detection of these inflammatory cells proved that delayed allograft rejection in recipients treated with anti-IL-17 mAb was correlated with decreased infiltration of inflammatory cells. All the above results indicate that IL-17 neutralization might delay the development of corneal allograft rejection by inhibiting inflammation, particularly neutrophil infiltration and that anti-IL-17 mAb has a protective effect on corneal rejection. Interestingly, neutralizing IL-17 antibody decreased the corneal injury in syngeneic transplantation, but inflammatory graft-infiltrating cells had no notable decrease at 14 days after transplantation. We attribute this to different timing of inflammatory cell detection. As a course of inflammation, syngeneic corneal transplantation did not reject. At the early stage of syngeneic transplantation, neutrophils were present and subsequently decreased with the disappearance of inflammation.

We also found that delayed allograft rejection was associated with decreased pro-inflammatory cytokines IFN-γ, IL-12p40, and IL-17 after IL-17 neutralization. Th1- and Th2-cell subsets appear to negatively regulate differentiation of Th17 cells [33]. IL-12 and IFN-γ have been found to suppress IL-17 production [34]. The addition of IL-12, IFN-γ, or IL-4 to cultures inhibits either IL-23- or TGF-β- and IL-6-stimulated differentiation of mouse and human Th17 cells [35-38]. IL-23, described as the pairing of the p19 subunit with the p40 subunit and shared with IL-12 [39], promotes IL-17 expression and the proliferation of IL-17-producing cells from a pool of memory T cells [34,40]. IL-12 is an important factor for Th1 cell development. These two related cytokines initiate and sustain Th1 cell-mediated immune responses [41]. However, in recipients treated with anti-IL-17 mAb, although IL-12p40 production decreased significantly, IL-23 production by splenocytes was minimal, indicating that IL-23-independent mechanisms may be responsible for the maintenance of the Th17 response [42]. In contrast, some papers have shown pathogenic roles of IFN-γ and IL-17 in corneal immune rejection of orthotopic allografts in mice and rats [27,42,43]. We hypothesize that reducing IFN-γ and IL-17 levels would block allograft rejection, which is consistent with our results in that delayed-rejected recipients with anti-IL-17 mAb treatment, IFN-γ and IL-17 production from splenocytes decreased significantly. Interestingly, IL-6 production was elevated after IL-17 neutralization, suggesting that IL-6 is upstream of IL-17 in the Th17 differentiation pathway. Production of Th2 cytokines IL-4 and IL-5 was also increased with IL-17 neutralization, indicating that IL-17 is critical for mediating corneal allograft rejection.

Of the large number of cell types analyzed for IL-17 expression, the CD4^+^ T cells have been previously reported to be the only producer of IL-17 [44-46]. However, recent reports have suggested that IL-17 is produced by Th17, natural killer, natural killer T, γδT, neutrophils, and CD8^+^ T cells [47-49]. In our study, we focused on Th17. In agreement with previous reports [50], we found that CD4^+^ T cells derived from spleens expressed IL-17 in allogeneic recipients, but there was very little or no IL-17 expression in syngeneic recipients and normal spleens at 14 days after transplantation. Th17-cell expression decreased with IL-17 neutralization, but there were no significant differences among control and anti-IL-17 mAb groups. Although pro-inflammatory cytokines that regulate Th17 cell differentiation changed correspondingly, IL-17 neutralization has no effect on the frequency of Th17 cells.

In summary, our results indicate that IL-17 neutralization significantly prolongs corneal allograft survival time and inhibits corneal allograft rejection to a certain extent by inhibiting production of graft-infiltrating inflammatory cells and decreasing secretion of pro-inflammatory cytokines. These findings may offer new treatment options for corneal rejection and other immune system transplantation concerns.
